# Does the Home-Visit Communicating Service by Postal Workers Improve the Mental Health of Older Persons Living Alone?

**DOI:** 10.1093/geroni/igaa057.976

**Published:** 2020-12-16

**Authors:** Hiroki Inagaki, Shuichi Awata

**Affiliations:** 1 Tokyo Metropolitan Institute of Gerontology, Itabashi-ku, Tokyo, Japan; 2 Tokyo Metropolitan Institute of Gerontology, itabasi-ku, Tokyo, Japan

## Abstract

In Japan, the number of older households living alone or married couples is increasing as society ages. For such households, Japan Post offers “Mimamori Home-visit service” a fee-based service where postal workers visit once a month to check their living and health conditions. We examined whether the use of this service improves the mental health of users. There were 10,592 service users as February 2019. The survey targeted 524 people (356 women) who started using the system in January or February (wave1) and continued using until August 2019 (wave2). The mean age was 79.5 years. Visiting postal workers conducted tablet-based interviews to assess social support networks (LSNS-6) and mental health (WHO-5). Information on gender, age, and family form (living alone or not) was provided by Japan Post. The WHO-5 average score was 16.4 for wave1 and 16.3 for wave2. Changes in mental health (WHO-5 scores) at 2 time points were compared by ANCOVA using family form (living alone or not) and social isolation (12 points or less/13 or more for LSNS-6) as explanatory variables and gender and age as covariates. The results showed a significant interaction between the 3 factors of time, family form, and social isolation. WHO-5 scores increased (14.2 to 15.3) in the group that lived alone and had 12 or less of LSNS-6. In the other group, the score was no change or lowered. It has been shown that mental health improves with the use of monitoring services in elderly people living alone and in social isolation.

